# ‘Life under the tent is not safe, especially for young women’: understanding intersectional violence among internally displaced youth in Leogane, Haiti

**DOI:** 10.1080/16549716.2017.1270816

**Published:** 2017-01-27

**Authors:** Carmen H. Logie, CarolAnn Daniel, Uzma Ahmed, Rebecca Lash

**Affiliations:** ^a^ Factor-Inwentash Faculty of Social Work, University of Toronto, Toronto, ON, Canada; ^b^ Women’s College Research Institute, Women’s College Hospital, University of Toronto, Toronto, ON, Canada; ^c^ Faculty of Social Work, Adelphi University, Long Island, NY, USA; ^d^ NEGES Foundation, Leogane, Haiti; ^e^ Faculty of Anthropology, Dalhousie University, Halifax, NS, Canada

**Keywords:** Displacement, gender-based violence, structural violence, masculinity, HIV

## Abstract

**Background**: Haiti’s 2010 earthquake devastated social, health, and economic infrastructure and left 2 million persons homeless. Over 6 years later 61,000 people remain displaced, most lacking protection, services, and durable solutions. Structural contexts elevate risks of gender-based violence (GBV) targeting internally displaced (ID) girls and women.

**Objective**: We used an intersectionality framework to explore lived experiences and understanding of violence among ID young men and women in Leogane, Haiti.

**Methods**: We conducted six focus groups, three with ID young women (n = 30) and three with ID young men (n = 30) aged 18–24 years, and 11 in-depth individual interviews with frontline workers in Leogane. Focus groups and interviews were conducted in Kreyol, transcribed verbatim, translated into English, and analyzed using narrative thematic techniques.

**Results**: Findings revealed violence experienced by ID youth was (re)produced at the intersection of gender, poverty, displacement, and age. Multi-level forms of violence included structural (e.g. poverty), community (e.g. gender norms, and interpersonal (e.g. family expectations) dimensions. Coping strategies spanned intrapersonal (hope), community (social support), and structural (employment/education) dimensions.

**Conclusions**: Interventions to reduce violence should be tailored to address the social inequities that emerge at the intersection of youth, poverty, displacement, and hegemonic gender norms.

## Background

Haiti’s 12 January 2010 earthquake led to the collapse of the country’s social, economic, and health infrastructure, resulting in the breakdown of community networks, increased poverty, and sexual violence [1–3]. One year after the earthquake, an estimated 2 million people were homeless [2]. Over 6 years later, approximately 61,000 persons remain displaced in Haiti, lack protection and basic services, and reside in informal settlements such as tents and camps [1]. Displacement elevates exposure to violence.

Research across the globe highlights the relationship between gender-based sexual violence (GBSV), conflict, and precarious living conditions among internally displaced persons. Research with youth [4,5] has explored the ways in which internally displaced youth in the United States and Colombia are vulnerable to violence due to the convergence of identity formation with the consequences of displacement. Less is known about internally displaced youths’ experiences of violence post-disaster, and in contexts of protracted displacement. Protracted displacement, persisting over 1 year with little to no progress, impacts over 700,000 persons in 34 global cases [1]. Knowledge gaps remain regarding youths’ lived experiences of protracted crises, and this information is needed to inform policies and programs.

Inadequate housing in the months and years after Haiti’s earthquake has produced additional survival challenges. The lack of housing after the earthquake resulted in young women exchanging sex for shelter [6,7]. Lack of security and protection in internally displaced persons camps, combined with inadequate lighting, bathing facilities, tents, and a lack of police patrols elevate exposure to violence – in particular GBSV – and precarious living for internally displaced persons in Haiti [1,2,8–10]. Moreover, a lack of parental support or disruption in living situations is linked to increased youth violence in Haitian communities [11]. Yet gender-based violence (GBV) has been a serious issue in Haiti for decades – even before the earthquake – shaped by contexts of poverty, ongoing civil and political conflict, and inequitable gender norms [12,13]. Nearly one-third of women prior to Haiti’s 2010 earthquake reported experiencing intimate partner violence (IPV) [14].

Intersecting factors increase gender inequities among internally displaced women in Haiti, including poverty, unemployment, insecure housing, and gender norms grounded in hegemonic masculinity [7,10]. Further, Haiti is the poorest country in the Western hemisphere with an unemployment rate predating the earthquake of 40% [15,16]. As of 2015, young Haitian women’s unemployment rate remained 5% higher than males [17]. Prior research in Haiti suggests that poverty may contribute to youths’ early sexual debut and engagement in transactional sex for survival [6,9].

Structural violence is a framework that has been applied to understand how social and structural contexts contribute to health inequalities and unequal life chances in Haiti [10,18]. These social contexts include inequitable gender norms, poverty, and lack of access to education; combined, these social, political, and economic forces cause harm to the body and to the soul [19] that compromises health and wellbeing. Literature has acknowledged the critical importance of understanding gender as intersectional and institutional [20], alongside other converging factors and identities that influence wellbeing. Applying an intersectional approach [21–24] to understand structural violence in post-earthquake Haiti involves exploring how multiple, intersecting axes of inequality – for example, based on gender, age, poverty, and displacement – produce both oppression and opportunity. Relational approaches [25] enhance understanding of the complexity of gender power dynamics, including diversity within genders, and the intersection of gender and other identities that produce different experiences of masculinity and femininity. For example, factors such as poverty can shape how masculinities are constructed and enacted [26–28]; gender hierarchies are produced in the construction of masculinities that contribute to GBV and compromise men and women’s health and wellbeing [27].

Gibbs, Sikweyiya, and Jewkes [27] discuss the importance of recognizing that poverty can create feelings of powerlessness, vulnerability, and despair, and GBV can be a method used by men who experience marginalization (e.g. through poverty) and do not experience the ideals of hegemonic masculinity to regain power and control. Limited research has explored intersectionality as a way to understand structural violence among internally displaced youth in disaster contexts such as post-earthquake Haiti. We aimed to fill gaps in knowledge about how youth at the nexus of protracted displacement perceive, experience, and respond to violence taking into account intersectional identities.

The study objective is to explore lived experiences and understanding of violence with internally displaced young men and women in Leogane, Haiti.

## Methods

### Setting

The study was conducted in Leogane, approximately 30 km from Port-au-Prince. Leogane was the epicenter of Haiti’s 2010 earthquake, and 80–90% of its buildings were destroyed, and one-quarter of its 120,000 population died, in the earthquake [29]. This was a community-based research project in collaboration with NEGES Foundation in Leogane; NEGES provide guidance into study objectives, methods, ethics, and implementation. The researchers CD and CHL, both female, had doctorates and were employed as faculty members in schools of social work in Canada and the United States at the time of the study. CD had several years of experience collaborating with NEGES in Leogane, and CD and CHL were conducting an HIV prevention intervention with internally displaced women with NEGES at the same time as this study. They both had experience and training in qualitative methods and working with youth. The researchers did not have a relationship with focus group (FG) participants prior to study commencement. Researchers had experience working with the key informants (KI) as community health workers and peer leaders in Leogane, and identified these persons as KI as they held expertise that was valuable in understanding lived experiences and realities of internally displaced youth in Leogane. Participants were informed of the background of the researchers and the purpose of conducting the research, that is, to better understand the lived experiences and priorities of internally displaced youth in Leogane.

### Participants

Eligibility criteria for FG participation included persons: aged 18–24 years, self-identified as internally displaced (living in a tent, camp, and/or residence due to being dislocated by the 2010 earthquake), and residing in Leogane or contiguous areas. We conducted 6 FG with a total of 60 internally displaced youth. This included 3 FG with internally displaced young men (age 18–19: n = 10; age 20–21: n = 10; age 22–24: n = 10) and 3 FG with internally displaced young women (age 18–19: n = 10; age 20–21: n = 10; age 22–24: n = 10). All participants were given an honorarium of $7.00 (USD) for their time and their transportation costs reimbursed. We hired six peer researcher assistants who were internally displaced youth; they included three young men (aged 18, 20, 23) and three young women (aged 18, 21, 24). We used peer-driven sampling methods [30,31], whereby peer researcher assistants invited youth from their social networks to participate in FG using snowball sampling and word-of-mouth techniques. Peer-driven sampling methods are appropriate for contexts where potential participants experience marginalization and populations may be harder to access [30,31]. To reduce bias we hired 6 peer researcher assistants of different ages, residence locations, and education levels, and limited to 10 the number of participants each peer researcher assistant could invite. All persons who were invited did participate in the study.

We conducted 11 KI in-depth individual interviews (IDII). KI were purposively selected for their knowledge of internally displaced youth, and included the peer research assistants described above (n = 6), three internally displaced community health workers working with NEGES, one community youth worker, and one research project coordinator.

### Data collection

FG typically lasted 60–75 minutes and IDII typically lasted 60 minutes; both were conducted in private rooms at NEGES in Kreyol and facilitated by study investigators (authors: CHL, CD) and a research assistant fluent in English and Kreyol who was also a translator. FG and IDII questions were informed by a semi-structured interview guide and sought to understand the lived experiences of violence among internally displaced youth in Leogane. FG and KI interview questions included: ‘We have heard a lot about violence, especially since the earthquake. What kind of things have you heard about violence targeting women? What are some causes of violence in your community?’

### Ethics

Research ethics approval was granted by Women’s College Hospital, University of Toronto, Toronto, Canada and Adelphi University, New York, USA. The informed consent was read aloud to all participants, participants provided written consent in Kreyol, and were offered a copy of the consent form.

### Analysis

All FG and interviews were digitally recorded in Kreyol, transcribed verbatim, and then translated into English. Field notes were made after the FG. KI were consulted to clarify transcript data, and findings were presented back to participants at youth community forums. Thematic analysis, a method used to identify, analyze, and report themes in the data, was employed to review the transcripts [32,33]. Two members of the research team thematically analyzed and coded the data using inductive approaches to explore new themes that emerged (e.g. hope, family expectations for girls to engage in sex work), as well as deductive approaches to identify themes from intersectionality, the guiding theoretical approach of the study (e.g. intersection of gender, age, and displacement). Member checking was conducted by sharing the findings with a co-investigator from the collaborating organization, the community youth worker, and research project coordinator to verify findings. We also shared findings with all of the youth participants at community forums in Leogane.

## Results

### Summary

Participant narratives articulated experiences of violence that were shaped by the intersection of social inequities associated with intersecting social identities of gender, poverty, displacement, and age. Violence was discussed at multiple levels: structural (poverty, unemployment, housing and food insecurity), community (gender norms, GBSV), and interpersonal (IPV, family expectations, survival sex work). Strategies for coping included intrapersonal (hope), community (social support), and structural (employment and education opportunities) dimensions.

### Intersection of housing insecurity and violence

Following the earthquake, most people in Leogane were displaced and lived in informal tent cities. Insecure tent housing was discussed as a site of violence. Participants reported many young women viewed insecure housing conditions as unsafe and making them easy targets of sexual violence. Most women expressed feelings of helplessness and being in a constant state of hypervigilance against sexual violence. Despite the presence of local authorities, participants noted sexual violence was commonplace and women were reluctant to report their assaults, as local authorities were believed to be ineffective at addressing these crimes. As one participant explained:

I want to tell the story of the life of people living under tents. Life under the tent is not safe, especially for young women. There was a young woman living under a tent alone, when three young men came in and violated her. After this happened, she wanted to take it to the police, however she was scared that they may come back and kill her. She was also discouraged because no one felt that the police would take charge. (FG young women aged 18–19)

A key informant noted the precariousness of living in tents that increases exposure to GBSV:

In the tent site the vagabonds do whatever they want. You may be sleeping here and your tent is getting unzipped. When you ask who he is, they pull a gun on you and you can’t do anything. (community health worker [CHW] 1)

### Intersection of poverty and GBV

Participants discussed how violence was a result of desperation from high rates of poverty and unemployment. Poverty and GBSV intersected as women discussed men may intend to conduct a robbery and, if women were present, may also perpetrate sexual violence:

Lack of money is the cause of the violence. A group of men can break into a house to steal, but when they get there they find women, they automatically add rape to their list of crimes, just because. Now the problem with housing. And now they are forced to sleep somewhere that is unsafe. (FG young women aged 20–21)

A key informant noted unemployment and insecure housing converged with a lack of law enforcement to produce GBSV:

When the earthquake just happened, all the time they are raping somebody or commit a violent act. Because there is no security in the tents. You may be in a tent and they set it on fire. There are no jobs. It is due to high unemployment rates. (CHW 3)

Persons living in poverty experiencing GBSV were discussed as less likely to receive justice:

I know a 17-year-old guy by my house that raped a 5-year-old. When the child’s mother came home she found her child covered in blood. The 17-year-old was at his house for 2 days, no one said anything and on the second day he saw the police outside his door ready to come get him. He spent 4 months in jail. He was in Leogane and then Ti Guave and after that you know, ‘deux cabrit maigre pas cuite’ (two skinny goats can’t cook), which means the 17-year old’s parents have a lot of contacts. The little girl’s parents don’t have any contacts. So with all these big heads involved, they simply have to shake the judge’s hand and ask him for what they want and need. So now this little girl’s life is taking a turn for the worse. (FG young men aged 20–21)

In addition to strangers perpetrating GBSV, participants also discussed how women’s poverty reduced their relationship power and enhanced vulnerability to IPV:

I think it’s mainly a relationship of power. Generally, the woman, even though they love someone, they will look for the person that can provide them security, food, housing. I think the insecurity here, it’s such a big deal, people, in general, but mostly women, since, in the culture it’s still the man that will provide everything for the family. The man is the one that works, gets the money. The women stay at home with the kids. There are a lot of women that get beaten up and they won’t tell anything because they don’t want the man to leave. It’s the same for the young people. (KI 1)

### Intersection of poverty, gender, and survival sex

Poverty contributed to young women’s engagement in survival sex work, including sex work for money, food, and/or education. A FG participant articulated:

In Leogane the young men do not respect women. There is economic hardship. There is no work and young girls do not go to school. She decides to sell her body so that she can eat or to make money to go to school. The men who have money want young girls. Because she is in need, whether she was interested in him or not she sleeps with him. (FG young women aged 20–21)

The above narrative elucidates the intersection of inequitable gender norms (‘the young men do not respect women’) and poverty that results in women and girls’ involvement in survival sex work. A youth peer leader further explained gender discrimination in the workforce that could lead to women’s sexual exploitation:

There could be something that the young people want but the parents don’t have the means to provide it, especially for the women. There could be a job posting and you go to an interview. You may have the skills to get the job but the boss says no, you must sleep with him first before he gives you the job. More men work than women. Now the women don’t have the possibilities. And they get abused in various ways. (Youth female peer leader, 24 years old)

A male FG participant discussed survival sex in the context of food insecurity:

Well it’s something that I can tell you that all Haitians already know. The major problem is hunger. Hunger. Once there is hunger in a country, the country will not function properly. The more a child doesn’t eat properly…. Sometimes, someone doesn’t need to know the person to offer him or her food. And just that can cause them to do things they don’t want to do, like have sex without condoms. And they could then catch HIV. (FG young men aged 20–21)

Another FG participant discussed survival sex work as a form of violence:

because you are not in a good economic situation, if the person does something for you, you are forced to sleep with him even if you did not want to. This is another type of violence because you did it, but did not do it voluntarily. (FG young women aged 22–24)

A male participant explained that gender norms around masculinity may contribute to men asking for sex as payment for gifts to women:

In my opinion, just because I gave a girl something I wouldn’t expect her to ‘pay me back’ by sleeping with me. And I also think that I could be in need and that the girl is the one that gets me out of trouble. If that was the case I wouldn’t feel required to do anything either. Some girls are like that. She will come by you with a problem and say she needs $50 for hair products or something. But the fact that she asked you, if you don’t do anything she’ll say that you’re gay. (FG young men aged 22–24)

This narrative also highlights a double standard, where a boy receiving a girl’s help will not be expected to repay the favor with sex. A key informant discussed family members’ involvement in having girls engage in survival sex to support the family:

I’ve heard of stories like that, young girls, that their own parents, their own father, will just ask them … not even ask them, just say, you do that, you’re going to sell your body so we can have some money. But the fact that there’s still a separation between what women can do and what men can do, and their role in the society, so that’s why, I think, you see that there are more girls that are selling their bodies or their services, as opposed to men, that will go work at more respectable jobs. They will risk themselves and health to help their family. (KI 2)

This narrative also highlights the gender inequities in society that constrain women’s employment opportunities and converge with women’s gender role expectations as caregivers.

### Coping strategies

Participants discussed multi-level coping strategies, including hope, social support, and education and employment opportunities.

### Hope

Participants discussed hope as a motivator for working towards goals. A young woman discussed how her hope helped her to prioritize a job over a relationship:

People are people but not everyone has the same objective. I want to accomplish something so I can help my entire family. I have always said that I will get in a relationship when I am ready, when I have accomplished certain things. I have a job then I can have a relationship. But there are young ladies who don’t see it like that, if a guy is talking to them, they answer. (FG young women aged 18–19)

Another participant discussed hope as a motivation for helping other young girls:

I will like to have the possibilities. In the same manner some older people used to help me and guide, I will like to do the same to the younger people. My hope comes from the way I live, the way that I understand life, the way I find people who give advice. My hope is someday I will help the young girls so maybe they will be able to change some aspects of their lives. (FG young women aged 22–24)

A young man discussed how his animal, representing his livelihood, helped to give him hope in reaching his goals for the future:

This is an ox that I have and that I love a lot. Because the way you see it now, he is how I will be earning some money tomorrow. All of my hope lies in him. I already told you that when I need to pay for school or school materials, it’s this little ox that makes it possible. (FG young men aged 18–19)

### Social support

Participants discussed how community support and reciprocity helped with coping and maintaining hope. A community health worker discussed the idea of reciprocity, being helped by others and helping others in return: ‘What really gives me hope? I always find people that encourage me. When I have, I share. I do good deeds. I help. While I am helping, I find those who also help me too’ (CHW 4).

A male youth leader also expressed the impact of community support on his life and willingness to help his community:

I can also help my community. I don’t exactly need to be a mayor to help my community; there are many other ways that I can help. I am an orphan living with people. That is why today I am proud. All these people, all the family welcomes me and appreciates me. (Youth male peer leader, 23 years old)

### Opportunity through education and employment

Participants expressed that education and employment were central to improving their lives and providing hope. A CHW situated their hope within access to education, employment, as well as faith: ‘School, work, and God give me hope. All that!’ (CHW 3). A FG participant discussed wanting HIV education to be shared with others, as well as the need for employment: ‘I would like to see what we learned here to be shared with others, I would like to see jobs’ (FG young women aged 22–24). Another youth leader discussed how support with her education helped her strive for her dreams: ‘I have hope because I believe in it first of all. I go to school and I have people who want to support me with my dreams’ (Youth female peer leader, 21 years old).

**Figure 1.**
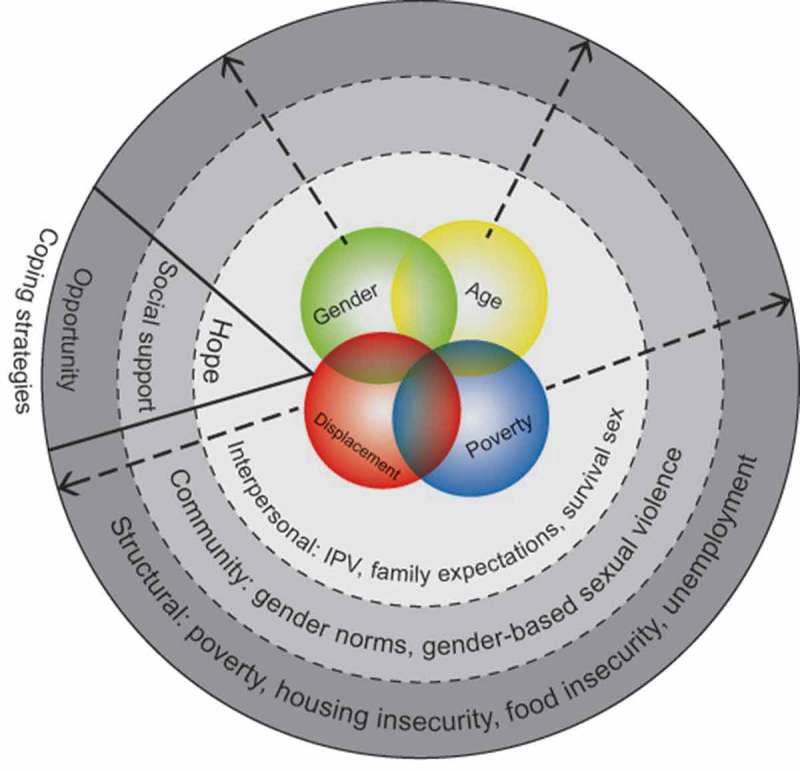
Conceptual model of intersectional violence and coping among internally displaced youth (n = 60) in Leogane, Haiti.

A conceptual framework of intersectional violence that integrates the themes in our analyses is presented in Figure 1. *Structural* (poverty, food and housing insecurity, unemployment), *community* (GBSV, gender norms), and *interpersonal* (IPV, family expectations, survival sex) dimensions of violence are represented as social ecological levels that connect with – and provide the context for – intersecting identities of youth, poverty, gender, and displacement. The overlapping circles for the identities reflect one of the central tenets of intersectionality, which is the interdependent and mutually constitutive nature of social identities [22,24,25]. The arrows radiating from the identities span all social ecological levels and are bidirectional, illustrating how structural, community, and interpersonal levels are interconnected. Intersectionality also highlights the opportunities that are produced at the intersection of identities [22], and the themes of hope (intrapersonal), social support (interpersonal), and opportunity (structural) reflect the strengths and resilience of internally displaced youth. This framework may be useful in future research focused on intersectional approaches to understanding violence.

## Discussion

Our study’s exploration of violence reveals that violence is situated at the intersection of gender dynamics, poverty, young age, and displacement for internally displaced young men and women in Haiti. Violence was discussed as complex and multi-level, operating across structural, community, and interpersonal levels. Our findings suggest that poverty is a significant underlying factor in contributing to violence; both young men and young women described desperation and unemployment as root causes of men’s involvement in GBV. Displacement resulted in precarious housing in tents; this increased young women’s exposure to GBSV. Extreme poverty and a lack of employment opportunities converged with gendered expectations for girls to support their family, and for girls to repay any assistance received from boys with sex: the urgent need to meet basic survival needs intersected with these gendered expectations to contribute to girls’ involvement in transactional sex.

A lack of educational and employment opportunities constrained youths’ ability to realize their potential: this reflects Galtung’s [34] conceptualization of structural violence. Participant narratives also portray the complexity of violence outlined in structural violence theory [34]. Types of violence participants reported included *direct* (e.g. sexual assault) and *indirect* (e.g. housing insecurity); these converged to produce *latent* violence, the pervasive feeling that danger and conflict could emerge at any time (e.g. fear of one tent’s being broken into, fear of assault). Narratives also highlighted *psychological violence* [34], or violence against one’s soul (e.g. feeling pressure to engage in transactional sex when given material goods, articulated by a participant thus: ‘you are forced to sleep with him even if you did not want to. This is another type of violence because you did it but did not do it voluntarily’).

Our findings corroborate literature that elucidates how social processes that (re)produce gender hierarchies are complicated by intersecting social positions such as poverty and age [27]. Young men living in poverty who do not hold the idealized and desirable forms of power represented in the construct of hegemonic masculinity may view violence as one of the only pathways to access power [25]. Our findings demonstrated that both internally displaced young men and women believed that it was desperation and poverty that fueled disenfranchised men targeting tent cities – precarious housing with limited security and protection – for robberies and GBSV. Violence in this context was a medium for internally displaced men with subordinated masculinities to access power through money and sex. Youth masculinity is constructed in relation to inclusion/exclusion from the capitalist economy [27]. Young men excluded from economic opportunities may seek out, and navigate, alternative ways of achieving masculinity and power, such as sexual violence [20,27]. Although men who are marginalized due to poverty and other social inequities experience subordinated masculinities, the ways in which they attempt to access power through violence in fact reinforce and support the inequitable gender hierarchies produced in hegemonic masculinities [25].

Research conducted before Haiti’s 2010 earthquake highlighted that men’s perpetration of IPV was associated with men’s unemployment, neighborhood poverty, and men’s endorsement of traditional gender roles, including a man’s right to beat his wife [14,35]. We found young internally displaced women at the intersection of displacement and precarious housing, extreme poverty, and inequitable gender norms, are at elevated risk of survival sex work, IPV, and GBSV. Their lives are further constrained by gender hierarchies that limit access to employment and education. This corroborates findings from research prior to Haiti’s earthquake that highlighted associations between women’s risk of IPV and not completing primary school, family contexts of IPV, and unequal relationship power[14,35], and post-earthquake research that situated women’s IPV risks in contexts of poverty and patriarchal gender norms [36]. This study contributes to the body of literature on internally displaced youths’ experiences post-disaster and in contexts of protracted crises by demonstrating the multi-level, intersectional nature of violence – as well as highlighting coping strategies that include hope, social support, and opportunity.

There are study limitations. We separated FG by gender to reduce the influence of hegemonic gender norms [36] on participant comfort level in discussions of gender roles, masculinity, femininity, and experiences of violence among young men or women. However, the authors’ female gender could have influenced data collection with the young men: they may have been able to speak more freely in a single-gender group with male facilitators. Second, the FG format for data collection may have provided limited opportunity for participants who were not comfortable speaking in groups to share experiences regarding violence and personal issues [37]. Future research could integrate more in-depth interviews with displaced youth to understand lived experiences of violence.

## Conclusion

Our findings have implications for multi-level interventions to reduce GBV among internally displaced youth. The Internal Displacement Monitoring Centre recommends that strategies with internally displaced people should focus on employment and income generation, as well as how displacement impacts social and psychological worlds [1]. Shelters and housing for internally displaced persons experiencing GBV have been called for [38]. The Inter-Agency Standing Committee Task Force on HIV in humanitarian settings has also highlighted the need to reduce GBV in HIV prevention programming [39]. Addressing structural contexts of poverty through interventions to build livelihoods among both men and women and provision of stable and secured housing are essential to tackling root causes of violence. For example, the IMAGE microfinance combined with gender intervention in South Africa was associated with reduced IPV [40]. Jewkes et al. [41] detail strategies to integrate hegemonic masculinity into interventions that engage men in challenging inequitable gender norms. For example, the Macho Factory in Sweden examines ways to change and challenge social norms of masculinity and violence, using techniques including films, exercises, and drama education [41]. Strengths that internally displaced youth identified as coping strategies – hope, social support, and striving for opportunity through education and employment – are considered resources that can be integrated into future intervention development. Taken together, these strategies hold the potential to address intersectional violence among internally displaced youth in Haiti.

## Paper context

Protracted displacement, persisting over 1 year with little progress, impacts over 700,000 persons in 34 global contexts. Knowledge gaps remain regarding youths’ lived experiences of protracted crises. This information can inform policies and programs. This qualitative study contributes to the literature on internally displaced youths’ experiences post-disaster and in contexts of protracted crises by demonstrating the multi-level, intersectional nature of violence – as well as highlighting coping strategies that include hope, social support, and opportunity.
